# Application of novel AI-based algorithms to biobank data: uncovering of new features and linear relationships

**DOI:** 10.3389/fmed.2023.1162808

**Published:** 2023-07-13

**Authors:** Lee Sherlock, Brendan R. Martin, Sinah Behsangar, K. H. Mok

**Affiliations:** ^1^Meta-Flux Ltd., Dublin, Ireland; ^2^Trinity Biomedical Sciences Institute (TBSI), School of Biochemistry and Immunology, Trinity College Dublin, The University of Dublin, Dublin, Ireland

**Keywords:** metabolomics, NMR, KarMeN, BATMAN, AI-based algorithm, lung cancer

## Abstract

We independently analyzed two large public domain datasets that contain ^1^H-NMR spectral data from lung cancer and sex studies. The biobanks were sourced from the Karlsruhe Metabolomics and Nutrition (KarMeN) study and Bayesian Automated Metabolite Analyzer for NMR data (BATMAN) study. Our approach of applying novel artificial intelligence (AI)-based algorithms to NMR is an attempt to globalize metabolomics and demonstrate its clinical applications. The intention of this study was to analyze the resulting spectra in the biobanks via AI application to demonstrate its clinical applications. This technique enables metabolite mapping in areas of localized enrichment as a measure of true activity while also allowing for the accurate categorization of phenotypes.

## 1. Introduction

**Figure 1:**
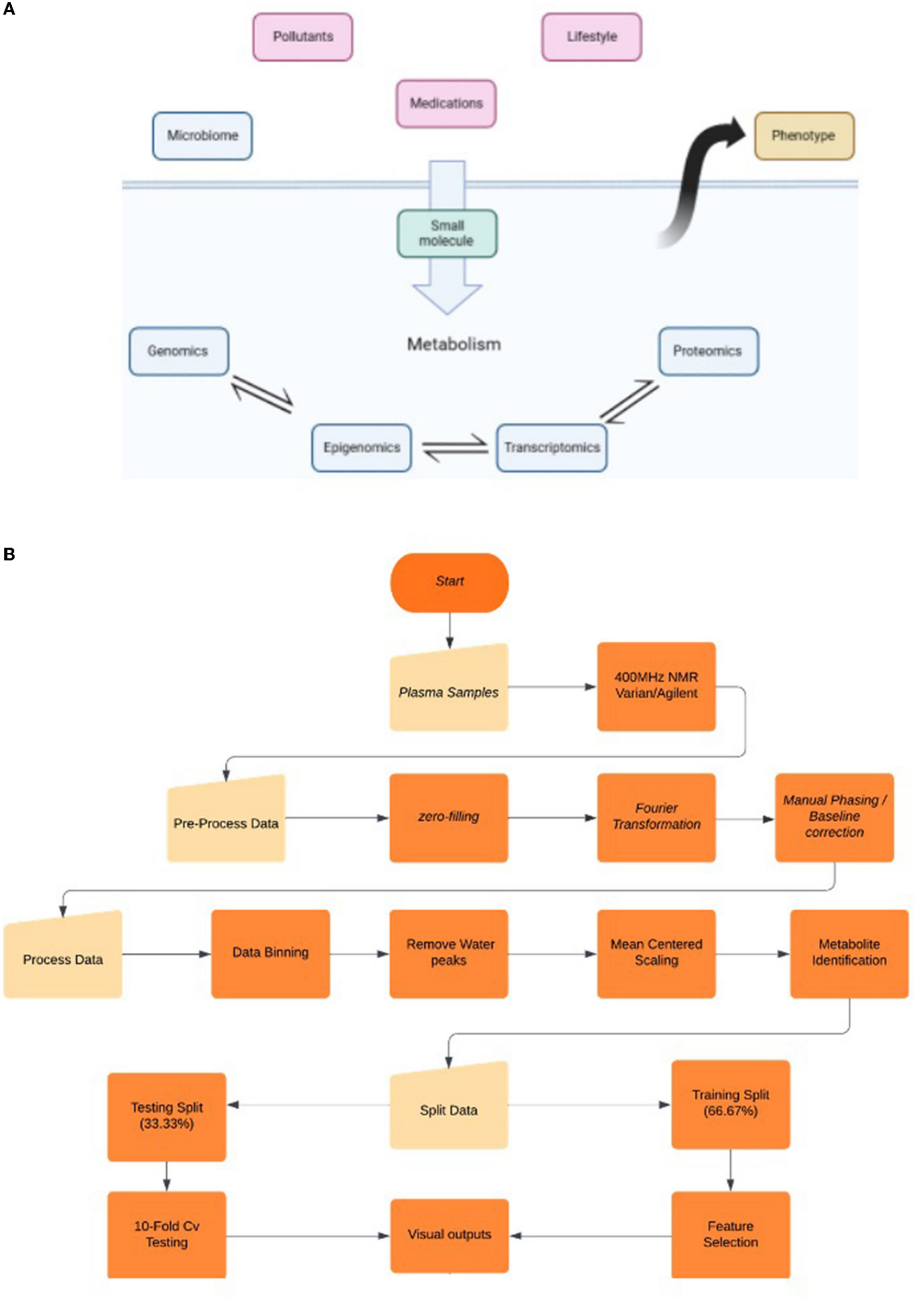
**(A)** Biological “omics” cascade and the factors that govern them. Targeted metabolomics focuses on the measurements of endogenous small molecules as a by-product of a metabolic pathway, while global metabolomics focuses on the fluctuation patterns and attributes said pattern to a pathway. Fluctuations in the “omics” cascade (blue layer) can be due to the influence of exogenous non-genetic factors (red) and can lead to alterations in phenotypes. Global metabolomics analysis can aid in the enhanced understanding of biomarkers/pathways and their correlation with etiology and diagnosis (6). **(B)** Workflow diagram highlighting the important milestones of the NMR and AI processes.

The field of metabolomics is the most recent addition to the “-Omics” discipline. The core objective of this emerging field is to record all metabolites within a biological sample. Metabolites are understood to be by-products of cellular metabolism with a weight of ~2 kDa or less (1, 2). Water-soluble metabolites have the ability to communicate with the environment and the microbiome due to the mobility around the open biological system (3). Consequently, metabolomics is essential for “systems biology” due to its particular scope analogous to fields such as genomics and proteomics (4). “Hence, genomics and proteomics identify what could happen, metabolomics identifies what is currently happening in a system” (5). The metabolomics framework is capable of examining endogenous metabolites and signal molecules that are by-products or participate in gene regulation, protein function, and enzymatic activity. Based on these, we identify ‘true activity' as a representation of what is currently happening in a biological system (5). Additionally, metabolomics is often a consequence of “exposomics”, which is a series of factors that include diet, lifestyle, pollutants, medication, and the microbiome itself (Figure 1A) (7). It is particularly valuable as it is capable of capturing the thousands of small molecule interactions within a given organism (8). Therefore, a significant portion of research has been invested in the potential of tracking the downregulation and upregulation patterns of metabolites or biomarkers in order to interpret fluctuations in biological function (9, 10).

Broadly speaking, there are two metabolomics methodologies: The first is targeted metabolomics, which establishes associations between defined metabolites and known phenotypic states (1). This approach remains to be desired as it requires a deep understanding of that pre-defined state and access to bioinformatic databases to cross-validate. Alternatively, untargeted metabolomics is the widening of the search for metabolites without prior knowledge of the state in question. This unbiased and semi-quantitative approach measures thousands of small molecules simultaneously with the core objective being the development of statistical and analytical methods that allow the tracking of entire metabolic pathways and fluctuation patterns (11–13).

A potential workhorse instrumentation for untargeted metabolomics integration is nuclear magnetic resonance (NMR) due to its holistic detection capability combined with high sensitivity (though not as high as mass spectrometry) for low molecular weight biomarkers. It is typical to use NMR and mass spectrometry (MS) in tandem with multivariate analysis (14). NMR spectroscopy is a technique that exploits atomic nuclei with non-zero magnetic moments to act as tiny probes for the detection of the local structure, dynamics, reaction state, and chemical environment within molecules. NMR spectra are unique, well-resolved, analytically tractable, and often highly predictable for small molecules. NMR analysis is, therefore, used for confirming the identity of a substance. Different functional groups are easily distinguishable, and identical functional groups with differing neighbors still give distinguishable signals. Following NMR's discovery in the 1940s, a plethora of new applications have emerged, and the technique has undergone major technological developments. NMR has now become an essential tool in the fields of chemistry, physics, biology, and medicine. Potential applications of this technology exist in multiple areas including structural biology, metabolomics, food science, toxicology, natural products research, pharmaceutical reaction and process monitoring, and organic chemistry (15–17). As NMR is inherently quantitative, its ability to determine metabolite concentrations in a reproducible manner allows it to serve as an additional variable of analysis for multiple phenotypes from a variety of biofluids.

In the case of NMR, the standardized workflow generates thousands of signals which include true signals from metabolites, adducts, and fragments, as well as noise signals from contaminants and artifacts (11, 12). Due to the sheer quantity of signals generated from a single NMR workflow, it is essential to develop tools that are capable of noise reduction, aiding in the analysis of “true signals,” allowing for more impactful outputs from downstream analysis. At present, there are issues regarding the scalability of technologies that are required to mainstream global metabolomics. Currently, there are software tools developed such as MVAPack, NMRProcFlow, and WorkFlow4Metabolomics. However, there are problems regarding the high-throughput applications of such software tools allowing for the development of artificial intelligence (AI) integration.

There is an abundance of applications that have demonstrated that AI is not a one size fits all; therefore, one must borrow and hybridize concepts from genome-wide association studies (GWASs) and Mummichog in an attempt to map all possible metabolite matches to a pathway via mass spectroscopy, solely focusing on regions of localized enrichment as they are assumed to be a reflection of “true activity” (18). Other methods include the Bayesian Automated Metabolite Analyzer for NMR (BATMAN) data approach, which performs spectral deconvolution using prior information on the spectral signatures of metabolites (19). When handling large metabolomic datasets, it is common to attempt to find meaning through multivariate analysis (MVA) methods such as principal component analysis (PCA) and partial least squares projection to latent structures (PLSs), all of which are attempts to segregate features that contribute to variation that are separated for further analysis, not too dissimilar from the mummichog approach (20). The recent integrations of AI into this space have seen the use of the least absolute shrinkage and selection operator (LASSO), PCA, self-organization maps (SOMs), and partial least square-discriminant analysis (PLS-DA) (8). AI is capable of identifying phenotypic variation via dimensional reduction, which indicates the biological pathway that differs among phenotypes and demonstrates the value and power these approaches have as they lend themselves to precision health (21).

Our approach involves harnessing global metabolomics in addition to multivariate analysis in tandem with NMR to investigate metabolites and their correlation with sex and lung cancer. In this study, we use the data provided by two large biobank databases. All data relating to sex were curated and analyzed by Rist et al. (22) and Bub et al. (23), while the lung cancer data were curated and analyzed by Padayachee et al. (19). The objective was to examine open-source datasets and apply our analytical techniques to observe variations and establish relationships in regions of localized enrichment. Regions of enrichment are then separated and probed for further correlations. Further probing defines the change in functional parameters induced via disease or aging. Upon examining the blood and urine, it became apparent that it was possible to identify patterns and classify participants in accordance to sex and lung cancer, with >90% accuracy.

## 2. Materials and methods

### 2.1. Data collection

For this investigation, we obtained open-source datasets from the health study by Rist et al. (22) and the lung cancer study by Padayachee et al. (19). In this study, we focused solely on the previously analyzed ^1^H-NMR spectra of blood plasma and urine samples obtained from lung cancer patients (*n*_cases_ = 69, *n*_control_ = 74) (19) and healthy men and women (*n* = 301) (23). Procedural steps differed per study; these include fasting periods, preparation, and storage of NMR sampling.

The KarMeN study (22, 23) recruited healthy men and women (+18 years old). In addition to blood and urine sampling (tested by NMR, GC-MS, and LC-MS), a variety of anthropomorphic measurements were taken but not utilized during our analysis. The sole features used for this study were the ^1^H NMR blood and urine analyses performed following a post-fasting period of 6 h, which meant that we were availing of only approximately 35% of the entire dataset provided by the study (22).

Padayachee et al. (19) collected previously analyzed data from lung cancer patients (*n*_case_= 69) from the Limburg Positron Emission Tomography Center (Hasselt, Belgium), while the control data (*n*_control_ = 74) were from Ziekenhuis Oost-Limburg (Genk, Belgium). Additional parameters of this study included: a 6-h fastening period, a glucose level of ≥ 200 mg/dl, and morning medication intake.

The strict inclusion/exclusion parameters and the handling of samples in both studies gave us confidence in the integrity and excellence of both datasets, thus enabling us to perform our own analysis. The inputs we availed of were solely that of ^1^H-NMR datasets.

### 2.2. Data processing

In one-dimensional ^1^H-NMR spectroscopy, the signals are represented as the frequency domain resulting from the Fourier transform of a time-domain signal. These are given in units of parts per million (ppm), which is pre-determined at 0.0 ppm based on the chemical shift reference. Data processing was performed prior to any analysis to ensure the integrity and reliability of the results.

For the Padayachee et al. (19) data, several pre-processing steps were conducted on the 400-MHz spectra using the Varian/Agilent software. These steps involved zero-filling and multiplication by an exponential apodization function of 0.7 Hz before Fourier transformation. Additionally, the spectra underwent manual phasing, automatic baseline correction using polynomials or splines, and referencing to trimethylsilyl-2,2,3,3-tetradeuteropropionic acid (TSP) at 0.015 ppm. The final pre-processing step involved normalizing the spectra by the total area under the curve, without accounting for the water and TSP signals.

Regarding the Rist et al. (22) data, both plasma and urine samples were subjected to untargeted NMR analysis using ^1^D ^1^H NMR spectroscopy. Plasma samples were measured at 310 K on an AVANCE II 600 MHz NMR spectrometer equipped with a 1H-BBI probehead and a BACS sample changer, while urine samples were analyzed at 300 K on a Bruker 600 MHz spectrometer equipped with either an AVANCE III with a 1H,13C,15N-TCI inversely detected cryoprobe or an AVANCE II with a 1H-BBI room temperature probe. The plasma spectra were referenced to the ethylenediaminetetraacetic (EDTA) acid signal at 2.5809 ppm and bucketed graphically, ensuring that each bucket contained only one signal or group of signals and no peaks were split between buckets. The urine spectra were resampled for a uniform frequency axis and aligned using “correlation optimized warping.” Subsequently, bucketing was performed using an in-house developed software based on Python, aiming to assign signals or groups of signals to individual buckets without splitting peaks between them. Finally, the resulting bucket tables were used for statistical analyses and machine learning algorithms.

Furthermore, the resulting pre-processing steps from the studies by Rist et al. (22) and Padayachee et al. (19) were subject to further investigation. The investigation of the above outputs was performed using Chenomx NMR Suite 8.1 (Chenomx, Edmonton, Canada) and Human Metabolome Database (HMDB) for the identification of metabolites. In addition, there were a variety of unknowns that could not be identified by harnessing either methodology. Therefore, the results section and corresponding graphs contain these unknown variables that can be identified as “Unknown – PPM”.

The data obtained from the study by Padayachee et al. (19) required further processing steps in an attempt to reduce the background noise and increase the overall resolution of the data. This was conducted by binning the data into further sub-intervals of 0.01 ppm. Conversely, the same approach could not be conducted on the data obtained from the study by Rist et al. (22) as the binning was conducted in-house and correlated with pre-defined metabolites. The difference in binning processes and MHz may be factors that allowed for variation in the results.

As per common practice in NMR, we removed water and its corresponding ppm as this often accounts for the majority of peak intensity and can mask minor variations in the NMR spectra. Due to the difference in obtained data, standardization was required, whereby the negative values within the dataset were set to zero and mean-centered scaling was applied to the Rist et al. (22) data. Feature values were transformed to follow a uniform or normal distribution for the Padayachee et al. (19) data. This helped to stabilize the variance and minimize the effects of outliers, resulting in improved performance of the predictive model. Scaling is important as it facilitates a fair comparison between different features.

Finally, the dataset was divided into two sets: a test set comprising 33% of the data and a training set with 66% of the data. This partitioning ensures an unbiased evaluation of the algorithm's performance. To determine the significance of different features in the dataset, the widely adopted statistical test known as the ANOVA *F*-test was employed for feature selection. In order to comprehensively evaluate the algorithm, a 10-fold cross-validation technique was applied. This method is commonly employed in machine learning to assess the algorithm's performance across multiple subsets of the dataset. By dividing the data into 10 equal parts, the algorithm was trained and evaluated 10 times, each time using a different combination of nine parts for training and one part for testing. This approach provides a more robust assessment of the algorithm's generalization capability and overall performance.

## 3. Results

The data were generated by obtaining open-source datasets from the Rist et al. (22) and Padayachee et al. (19) lung cancer studies. In this study, we focused solely on the previously analyzed ^1^H-NMR spectra of blood plasma and urine samples obtained from lung cancer patients (*n*_cases_ = 69, *n*_control_ = 74) (19) and healthy men and women (*n* = 301) (23). The data were structured and analyzed using our own in-house artificial intelligence (AI) and machine learning (ML) combined with classic statistical approaches to isolate features of interest and hone in on localized regions of enrichment for further analysis and to correlate said features with individual metabolites and extrapolate for metabolites that are predictive of phenotypes of interest. The analysis in this section was performed via global metabolomics, which demonstrates simultaneous analyses of multiple features to categorize a phenotype of interest. The figures below show heatmaps, minimum spanning trees, boxplots, volcano plots, and PLS to demonstrate the phenotypic categorization, which lends itself to clinical capabilities.

We tested the integrity of our outputs by comparing them to the published analyses of the original datasets (19, 22). The mean specificity - which describes the amount of correctly predicted positives or “regions of enrichment” - we obtained was 0.97 for the KarMeN study (22) and 0.93 when distinguishing lung cancer of Padayachee et al. (19). Additionally, the precision of the model, which describes the portion of true positives among actual positives, was measured to be 0.96 in KarMeN and 0.93 in the Padayachee et al. study. The above statistics can be represented on a scale of 0–1, where 0 represents poor performance and 1 perfect performance.

### 3.1. Lung cancer case study

**Figure 2:**
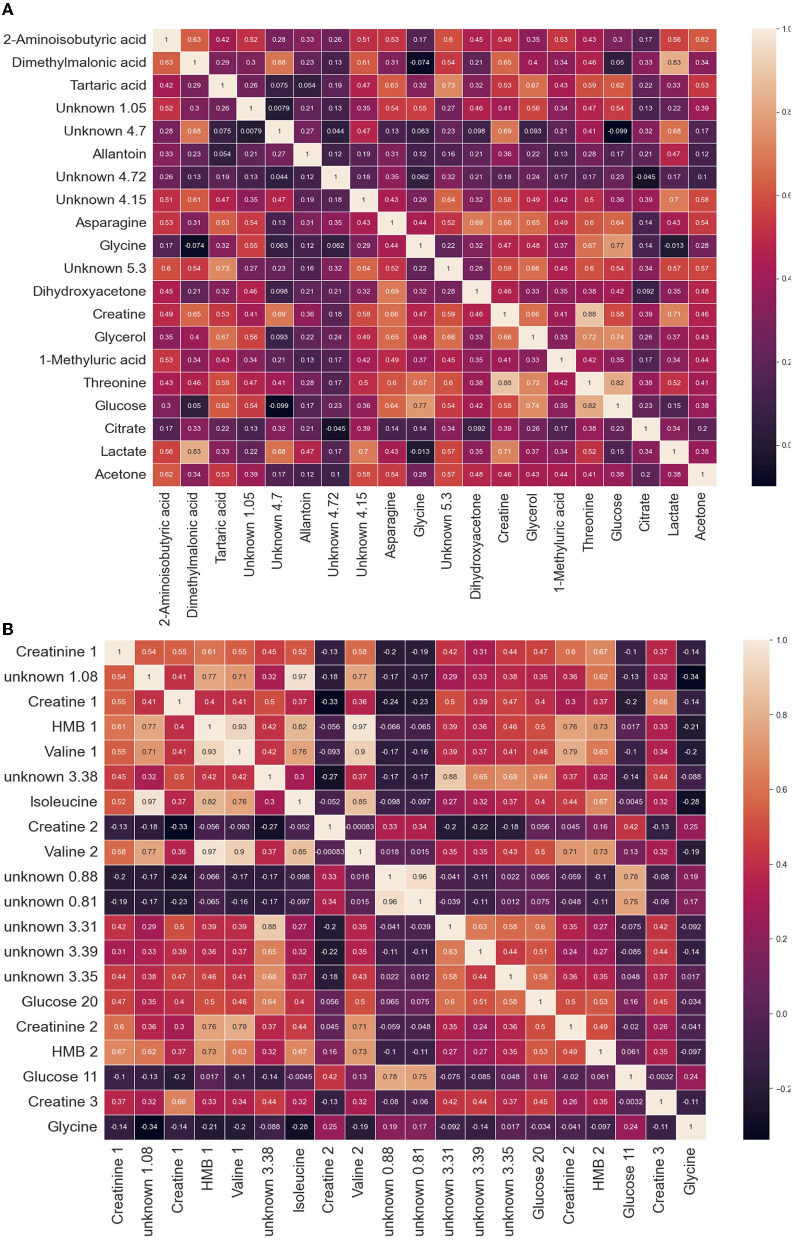
Heatmap of leading features in **(A)** lung cancer cohorts and in **(B)** health and sexes. This heatmap is a representation of the top features and the correlations relative to other features. The feature was determined by a singular NMR unit (bin or bucket), measured in units of chemical shift (ppm). The location of the ppm was determined by ANOVA F-values. The features found through NMR analysis of plasma can be used to categorize the **(A)** lung cancer metabolome and **(B)** among sexes and determine the states of health.

**Figure 3:**
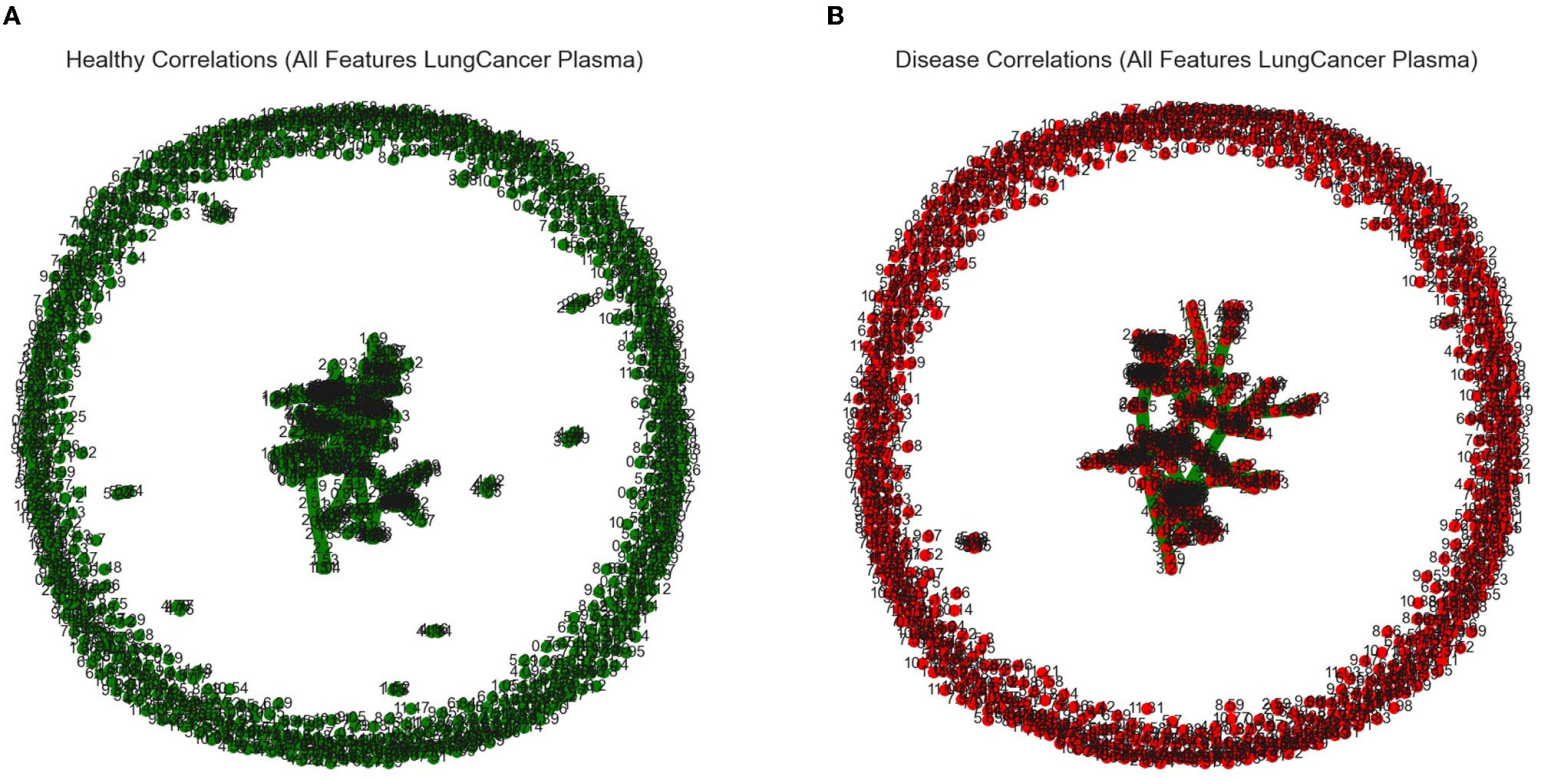
Graphical outputs visualizing the linear relationship between ppm. **(A)** Minimum spanning tree (Mst) generated by the Fruchterman–Reingold algorithm used to visualize all ppm in the healthy category with correlations above a 90% threshold. Nodes closer together in the center have a stronger correlation and nodes far apart around the perimeter have little to no correlation. **(B)** Mst used to visualize all ppm in the diseased category with correlations above a 90% threshold.

**Figure 4:**
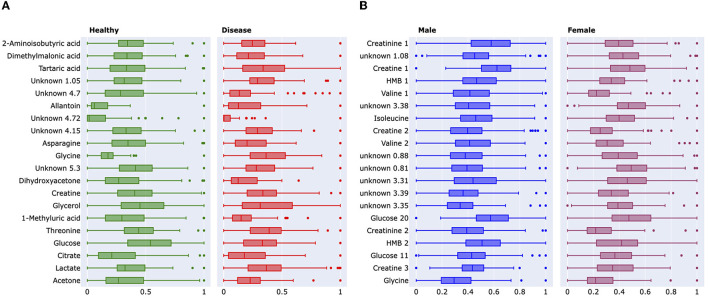
**(A)** Boxplots demonstrating the significance of changes between healthy controls and lung cancer groups and between male–female cohorts. The boxplot demonstrates the absolute difference between the means of each feature. These features were further analyzed and identified to be the following metabolites; 2-aminoisobutyric acid, dimethylmalonic acid, tartaric acid, and glycine. These identified metabolites were among the lead features used to categorize the phenotypes of interest. Green represents the healthy controlled cohort, while red represents the lung cancer cohort. The binned NMR spectral data from the Padayachee et al. (19) study were used to generate these graphs. **(B)** Boxplot demonstrates the absolute difference of the means of each feature. These features were further analyzed and identified to be the following metabolites; creatinine 1, creatine 1, 2-hydroxy-2-methylbutyric (HMB) acid, valine 1, valine 2, isoleucine, and glucose 20. These identified metabolites were among the lead features used to categorize the phenotypes of interest, while other points of interest include U 0.88 ppm and U 1.08 ppm. Blue represents the male cohort, while purple represents the female cohort. The binned Plasma NMR spectral data from the Bub et al. study were used to generate these graphs.

**Figure 5:**
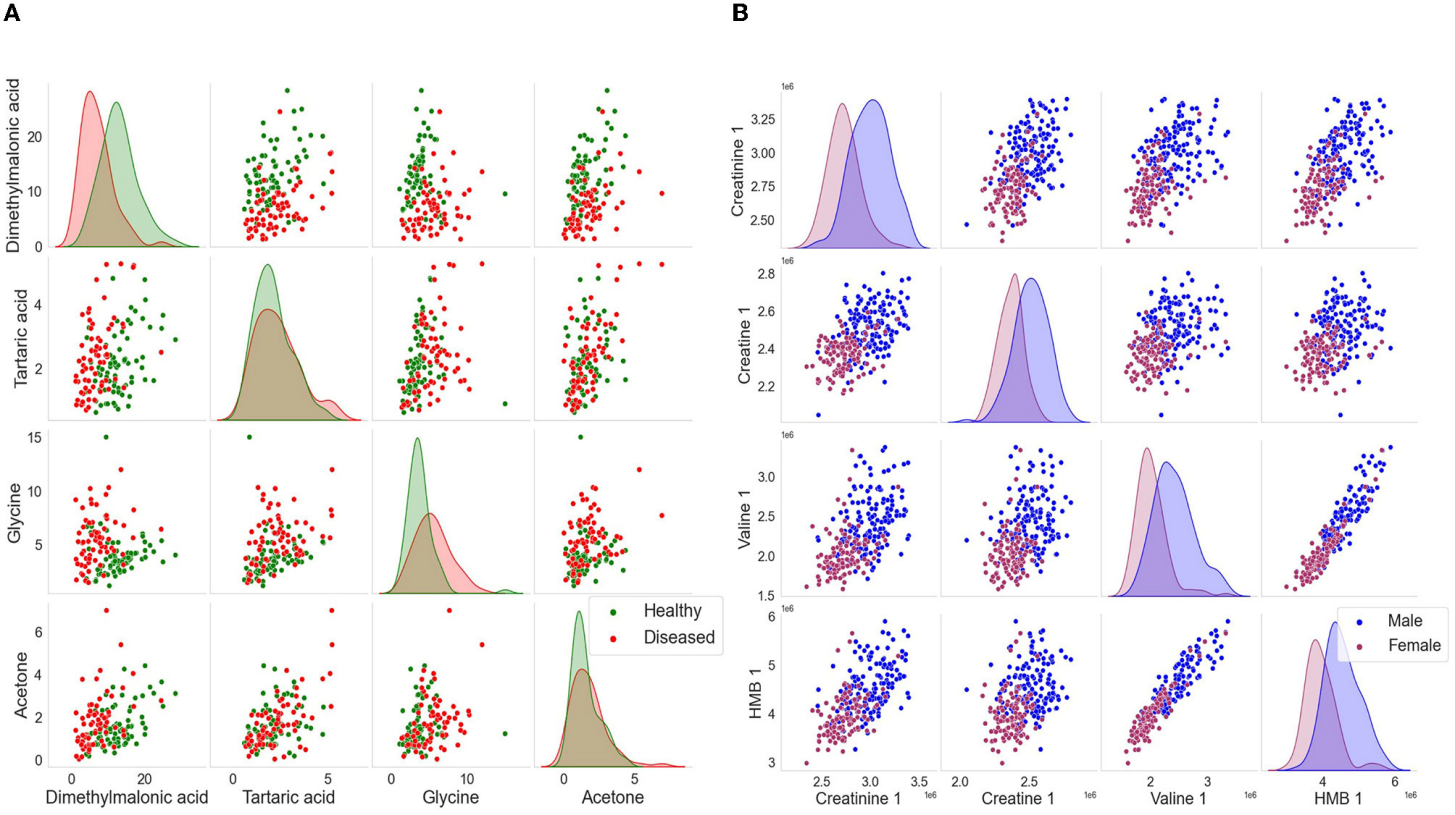
Kernel density plot used to visualize the distribution of lung cancer and the distribution of male–female cohorts. The above scatter plots demonstrate a clear separation among the cohorts. **(A)** For the lung cancer cohorts, the features of interest include dimethylmalonic, tartaric acid, glycine, and acetone. **(B)** For the distribution of sexes, the features of interest include creatinine 1, creatine 1, 2-hydroxy-2-methylbutyric (HMB) acid, and valine 1.

**Figure 6:**
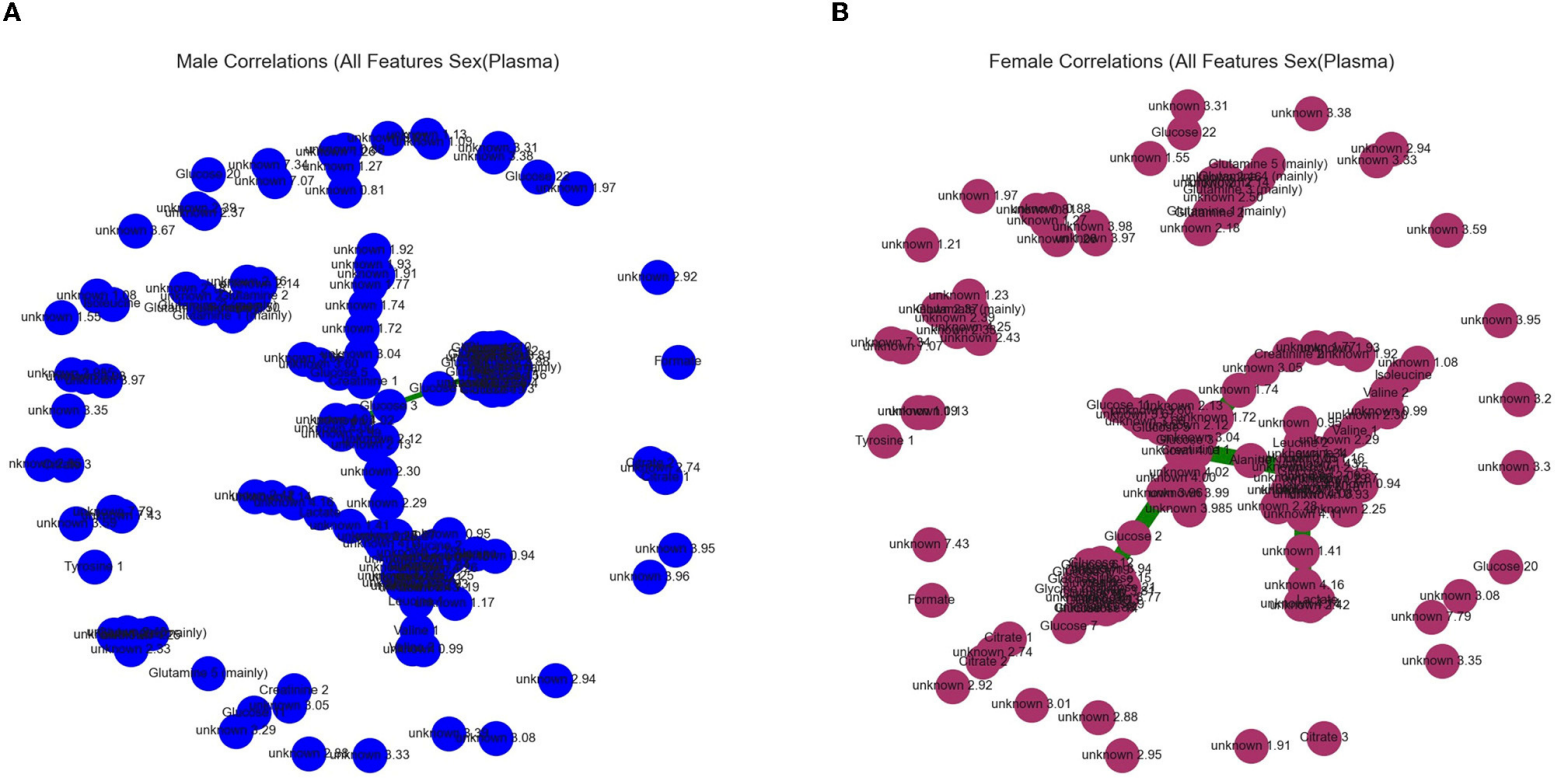
Graphical outputs visualizing the linear relationship between ppm. **(A)** Minimum spanning tree (Mst) generated by the Fruchterman–Reingold algorithm used to visualize all ppm in the male (blue) category with correlations above a 90% threshold. Nodes closer together in the center have a stronger correlation and nodes far apart around the perimeter have little to no correlation. **(B)** Mst used to visualize all ppm in the female (purple) category with correlations above a 90% threshold.

Our analysis of the data provided from the Bayesian Automated Metabolite Analyzer lung cancer study (19) yielded an overall 0.92 accuracy, with a mean specificity of 0.90 and a mean sensitivity of 0.93. The healthy precision value was 0.93, with a recall of 0.91 and an f1-score of 0.92. For the disease precision, it was 0.90, with a recall of 0.93 and an f1-score of 0.91. The area under the receiver operating characteristic curve (AUC-ROC) is calculated by plotting the true positive rate against false positive, where 1 represents perfect and 0.5 worst. The Padayachee et al. (19) data had an AUC-ROC of 0.92 (Figures 2–6).

Figure 2A is a heatmap of leading features in lung cancer cohorts. The leading 20 metabolites contained in this heatmap are essential for characterizing phenotypic states. Of these 20, we have found asparagine, creatine, glycerol, threonine, glucose, citrate, and lactate. Moreover, we have identified tartaric acid, which was not on the list of key metabolites in the Padayachee et al. (19) study. Interestingly, tartaric acid is known as a lung cancer biomarker and can be found in HMDB (24).

Our *in silico* analysis provided the following: Figures 3A and B are graphical outputs to visualize metabolomic relationships distilled down from a total of approximately 2 million relationships. The distillation of these relationships is further represented in Figures 4A and 5A which highlight the variability in the top-ranking metabolites. In summary, we have funneled down the key metabolites involved in lung cancer.

### 3.2. KarMeN health analysis among sexes

Our analysis of the data provided from the Karlsruhe Metabolomics and Nutrition study (22, 23) predicted sex solely using ^1^H-NMR data derived from plasma, yielding an overall accuracy of 0.95, with a mean specificity of 0.97 and a mean sensitivity of 0.92. The male precision value was 0.95, with a recall of 0.97 and an f1-score of 0.96. For the female precision, it was 0.96, with a recall of 0.93 and an f1-score of 0.94. The AUC-ROC was computed to be 0.95 (Figures 2–6).

Figure 2B is a heatmap of leading features in the determination of sex in healthy cohorts. The leading 20 metabolites contained in this heatmap are essential for characterizing phenotypic states. Of these 20, we have found creatinine, creatine, glycerol, glycine, sarcosine, isoleucine, and valine. Moreover, we have identified 2-hydroxy-2-methylbutyric (HMB) acid, which was not in the list of key metabolites in the Rist et al. (22) study.

Figures 6A and B are graphical outputs to visualize metabolomic relationships distilled down from a total of approximately 2 million relationships. The distillation of these relationships is further represented in Figures 4B, 5B, which highlight the variability in the top-ranking metabolites. In summary, we have funneled down the key metabolites involved in distinguishing sex in healthy people.

## 4. Discussion

The primary objective of this study was to analyze the human metabolome in the plasma by way of globalized metabolomics profiling by harnessing ^1^H-NMR, to determine the factors that significantly impact the metabolic profile of a healthy cohort compared to a lung cancer cohort, and to distinguish the variables among the sexes. Therefore, we performed our study and established a strict *in silico* experimental standardization, which we applied to data structuring, data treatment, and post-analysis treatments. When collecting open-source data, we ensured that all sample collections were standardized in terms of fasting, collection time points, and general pre-analysis handling. We also searched for healthy datasets with strict exclusion and inclusion criteria that excluded groups that suffered from acute or chronic diseases or were on medication, as we wanted a dataset that represented “true health,” thereby decreasing variation. In contrast, the medication and acute/chronic disease exclusion criteria cannot be applied to the lung cancer cohort as they must undergo medical treatment in tandem with the study. Furthermore, this fundamental difference may be one variable that explains the variability when testing the integrity of the algorithm. Through additional analysis, we found that our process is capable of generating high-integrity categorization with minimal variation. The difference among predictive capabilities per dataset could be due to the number of samples; *n* = 301 (22) and *n* = 143 (19). More specifically, Rist et al. (22) binned 138 sex features as pre-determined metabolites, while 1,134 features were binned as 0.01 ppm increments in the data of Padayachee et al. (19).

Furthermore, some AI algorithms may require a relatively small amount of data to achieve satisfactory results, while others, particularly deep learning algorithms, often benefit from large-scale datasets. The size of the dataset required is directly proportional to the type of AI used and its field of application. Even a large dataset may not be useful if it is noisy, incomplete, or biased. A primary issue is the problem of complex, highly specialized, and specific fields focusing on molecular interactions, protein structures, or drug discovery that typically require domain expertise and specialized knowledge. As a result, the problem space is more constrained, and the available data may be more targeted and focused. In such cases, a smaller sample size can still provide meaningful insights and accurate predictions.

The impact of our analytical approach can be found in Figure 4. Many of our leading 20 metabolites have significant overlap with the pre-existing analysis (19, 22). Along with these, we have uncovered previously unidentified metabolites, such as tartaric acid and 2-hydroxy-2-methylbutyric acid (HMB), in lung cancer and sex identification, respectively (22, 24). We wish to emphasize that Rist et al. utilized clinical chemistry, liquid chromatography, and mass spectrometry along with NMR spectroscopy to identify the top metabolites. However, our analysis only required one-third of the original dataset, and we only utilized the NMR dataset. Despite this, our analysis has uncovered not only similar metabolites but also those which are unique.

We recognize that there are requirements for additional analysis and broadening of the inclusion criteria. Participants that are obese and/or smoking must be included and recorded for an accurate representation of the healthy population, as studies demonstrate that nicotine does have neuroprotective qualities (25); therefore, we can assume their metabolic profile would be variable. We also need to recognize the influence of “exposomics” and how it can greatly influence the “omics” cascade, especially those that are variable per region, such as carcinogens and diet (Figure 1A) (6).

Owing to the fact that NMR metabolomics provides a quantitative and holistic view of all of the metabolites contained, there is no reason that this technology cannot be applied to other diseases. In this article, we have successfully harnessed AI and metabolomic techniques to broaden the search parameters that aid in a comprehensive understanding of disease and wellbeing. The advancements made here can offer a snapshot of the entire biological system, which allows us to ascertain an accurate understanding of the phenotype in question, paving the way for true precision medicine.

## 5. Conclusion

From our analyses of NMR spectra from two separate biobanks, we have established that our approach has direct clinical applications. Our approach of harnessing AI and NMR to globalize metabolomics enables us to identify metabolites, to highlight them as regions of localized enrichment as a measure of true activity, while enabling us to accurately categorize phenotypes of interest.

## Data availability statement

Publicly available datasets were analyzed in this study. This data can be found at: Padayachee et al. (19) and Rist et al. (22). Subsequently, the data was formatted, converted and processed, and are made available in this publication.

## Ethics statement

Ethical review and approval was not required for the study on human participants in accordance with the local legislation and institutional requirements. The patients/participants provided their written informed consent to participate in this study.

## Author contributions

LS and KHM initially discussed the potential of this research. LS, BM, and SB were involved in the coding and statistical evaluation of the data. LS, BM, and KHM wrote the manuscript. All authors contributed to the article and approved the submitted version.
