# Supplementary material for: Application of novel AI-based algorithms to biobank data: uncovering of new features and linear relationships
Source: Front Med (Lausanne). 2023 Jul 13;10:1162808. doi: 10.3389/fmed.2023.1162808 (PMC10373878; doi:10.3389/fmed.2023.1162808)
Supplement: Supplementary file 1 [file Table_1.pdf]

Table 1. A list of the top 20 features identified in the lung cancer dataset. The median intensity is provided for both the healthy control and case.

Metabolite	HMDB	Median healthy intensity	Median case intensity
2-Aminoisobutyric acid	HMDB01906	0.344368524	0.246269303
Dimethylmalonic acid	HMDB02001	0.340212425	0.212220739
Tartaric acid	HMDB00956	0.336417982	0.338111206
Unknown 1.05	N/A	0.316116651	0.289703445
Unknown 4.7	N/A	0.285145482	0.133864287
Allantoin	HMDB00462	0.060525077	0.126908694
Unknown 4.72	N/A	0.015752133	0.001141043
Unknown 4.15	N/A	0.332087244	0.288869578
Asparagine	HMDB33780	0.348407428	0.200346401
Glycine	HMDB00123	0.177044788	0.363575333
Unknown 5.3	N/A	0.412876099	0.280011389
Dihydroxyacetone	HMDB01882	0.268441398	0.124489187
Creatine	HMDB00064	0.405938128	0.336335524
Glycerol	HMDB00131	0.450831359	0.313484022
1-Methyluric acid	HMDB0003099	0.296189046	0.149808915
Threonine	HMDB00167	0.440027044	0.389729605
Glucose	HMDB0000516	0.541368443	0.332530039
Citrate	HMDB00094	0.209559251	0.177567237
Lactate	HMDB0003328	0.323417187	0.365906611
Acetone	HMDB01659	0.266631955	0.227400906

Table 2. A list of the top 20 features identified in the sex (plasma) dataset. The median intensity is provided for both Male and Female.

Metabolite	HMDB	Median male intensity	Median female intensity
Creatinine 1	HMDB00562	0.578903076	0.395399319
unknown 1.08	N/A	0.451796675	0.428062597
Creatine 1	HMDB00064	0.623770634	0.482217385
HMB 1	HMDB01987	0.466476042	0.337528979
Valine 1	HMDB0034366	0.414484182	0.223660915
unknown 3.38	N/A	0.404949044	0.470481354
Isoleucine	HMDB00172	0.459669519	0.398653666
Creatine 2	HMDB00064	0.396731954	0.25258421
Valine 2	HMDB0034366	0.412497841	0.306450289
unknown 0.88	N/A	0.379148905	0.392334179
unknown 0.81	N/A	0.392272613	0.491986458
unknown 3.31	N/A	0.438859247	0.462019444
unknown 3.39	N/A	0.36200012	0.337984265
unknown 3.35	N/A	0.339608248	0.389482268
Glucose 20	HMDB0000516	0.574538747	0.474087273
Creatinine 2	HMDB00562	0.390534218	0.217924925
HMB 2	HMDB01987	0.508423899	0.41772926
Glucose 11	HMDB0000516	0.428429505	0.365845914
Creatine 3	HMDB00064	0.433893842	0.348360038
Glycine	HMDB00123	0.292320381	0.217176177
